# Transcriptional Targeting of Primary and Metastatic Tumor Neovasculature by an Adenoviral Type 5 Roundabout4 Vector in Mice

**DOI:** 10.1371/journal.pone.0083933

**Published:** 2013-12-23

**Authors:** Zhi Hong Lu, Sergey Kaliberov, Rebecca E. Sohn, Lyudmila Kaliberova, David T. Curiel, Jeffrey M. Arbeit

**Affiliations:** 1 Urology Division and Department of Surgery, Washington University in St. Louis Medical School, St. Louis, Missouri, United States of America; 2 Department of Radiation Oncology and Biological Therapeutics Center, Washington University in St. Louis Medical School, St. Louis, Missouri, United States of America; 3 Siteman Cancer Center, Washington University in St. Louis Medical School, St. Louis, Missouri, United States of America; 4 Cell Biology Department, Washington University in St. Louis Medical School, St. Louis, Missouri, United States of America; Medical Center at Seattle, United States of America

## Abstract

New approaches targeting metastatic neovasculature are needed. Payload capacity, cellular transduction efficiency, and first-pass cellular uptake following systemic vector administration, motivates persistent interest in tumor vascular endothelial cell (EC) adenoviral (Ad) vector targeting. While EC transductional and transcriptional targeting has been accomplished, vector administration approaches of limited clinical utility, lack of tumor-wide EC expression quantification, and failure to address avid liver sequestration, challenged prior work. Here, we intravenously injected an Ad vector containing 3 kb of the human roundabout4 (*ROBO4*) enhancer/promoter transcriptionally regulating an enhanced green fluorescent protein (EGFP) reporter into immunodeficient mice bearing 786-O renal cell carcinoma subcutaneous (SC) xenografts and kidney orthotopic (KO) tumors. Initial experiments performed in human coxsackie virus and adenovirus receptor (*hCAR*) transgenic:*Rag2* knockout mice revealed multiple ECs with high-level Ad5ROBO4-EGFP expression throughout KO and SC tumors. In contrast, Ad5CMV-EGFP was sporadically expressed in a few tumor vascular ECs and stromal cells. As the *hCAR* transgene also facilitated Ad5ROBO4 and control Ad5CMV vector EC expression in multiple host organs, follow-on experiments engaged warfarin-mediated liver vector detargeting in *hCAR* non-transgenic mice. Ad5ROBO4-mediated EC expression was undetectable in most host organs, while the frequencies of vector expressing intratumoral vessels and whole tumor EGFP protein levels remained elevated. In contrast, AdCMV vector expression was only detectable in one or two stromal cells throughout the whole tumor. The Ad5ROBO4 vector, in conjunction with liver detargeting, provides tractable genetic access for in-vivo EC genetic engineering in malignancies.

## Introduction

The tumor neovascularization field remains challenged by the multiple evasion mechanisms induced in malignancies during antiangiogenic therapies [1]. The discovery of vascular endothelial growth factor (VEGF) [2] and its delineation as one of the predominant tumor produced angiogenic factors spawned a plethora of drugs and biologics targeting tumor production, stromal availability, and VEGF receptor signal transduction [3]. Despite impressive tumor size reductions in some patients, tumor growth eventually resumes. De novo or acquired tumor antiangiogenic therapy resistance is due to several factors. One evasion mechanism is cancer cell production of untargeted angiogenic factors [1]. Another mechanism is tumor chemo- and cytokine endocrine secretion mobilizing and recruiting proangiogenic bone marrow myeloid and immune cells [4]. A related mechanism is production of untargeted angiogenic factors by tumor-activated stromal fibroblasts [5]. Finally, tumors can shift their growth patterns and invade deeply into tissues by host blood vessel cooption [6].

While the principal function of tumor vasculature was presumed to be a conduit for nutrient and oxygen influx and metabolic efflux, emerging studies demonstrated that the microvasculature and the vascular endothelial cell (EC), are crucial components for establishment and maintenance of niches for host organ stem cells [7]. Tumor stem/initiating cells have also been identified in these perivascular niches [8]. This perivascular niche is maintained by short range, “angiocrine”, EC growth factor secretion and direct contact between tumor cells and host microvessels [9]. Angiocrine niche functions could be responsible for angiogenic inhibitor resistance and provide a permissive focal microenvironment for selection of aggressive tumor emergence [9].

These multifaceted resistance mechanisms have fostered renewed interest in targeting tumor EC signaling pathways that encompass both angiocrine and perfusion functions. Adenovirus (Ad) is one potential delivery vehicle for tumor EC targeting [10], [11]. Systemic injection of EC targeted Ads circumvents the recurring challenge of tumor permeation vexing local vector injection, and addresses the ultimate challenge of multiorgan metastatic disease. However, prior studies failed to investigate vector vascular expression in an extensive panel of host organs, and elucidate global determination of reporter expression distribution throughout the tumor neovasculature. Here we took initial steps toward implementation of endothelial targeting using a first generation adenovirus serotype 5 (Ad5) vector. We engaged a transcriptional targeting strategy, creating a vector whose reporter gene was regulated by the endothelial predominant human roundabout4 (*ROBO4*) enhancer/promoter [12]. In hypervascular 786-O renal carcinoma xenografts, orthotopic tumors, and spontaneous metastasis, Ad5ROBO4 directed enhanced green fluorescent protein (EGFP) expression to the neovasculature, whereas a vector whose reporter was controlled by the human cytomegalovirus (CMV) enhancer/promoter produced sporadic EC reporter expression in only one or two vessels throughout the tumors. Ad5ROBO4 is the first step towards a portfolio of vectors with the capacity for genetic manipulation of tumor ECs to disrupt the ability of the microenvironment to support tumor growth and therapy resistance.

## Methods

### Adenoviral vector construction

Replication incompetent *E1*- and *E3*-deleted Ad5CMV-EGFP and Ad5-ROBO4-EGFP vectors were created using a two-plasmid rescue method. The plasmids encoded expression cassettes comprised of the human cytomegalovirus (CMV) major immediate-early promoter/enhancer or the *ROBO4* enhancer/promoter elements coupled to the enhanced green fluorescent protein *EGFP* gene, followed by the bovine growth hormone polyadenylation signal. These expression cassettes were cloned into a shuttle plasmid (pShuttle, Qbiogene, Carlsbad, CA) and confirmed using restriction enzyme mapping and partial sequence analysis. The shuttle plasmids were linearized with *Pme* I enzyme and integrated into the Ad5 genome by homologous recombination with pAdEasy-1 plasmid in *E. coli* strain BJ5183. Recombinant viral genomes were transfected into HEK293 cells using SuperFect Transfection Reagent (QIAGEN, Chatsworth, CA), where they were packaged into virus particles. Ad5CMV-EGFP and Ad5ROBO4-EGFP were propagated in HEK293 cells, purified twice by CsCl gradient centrifugation and dialyzed against 10 mM HEPES, 1 mM MgCl_2_, pH 7.8 with 10% glycerol. The viral particle (vp) concentration was determined by absorbance of dissociated virus at A_260_ nm using a conversion factor of 1.1×10^12^ vp per absorbance unit. Viral titer also was measured by a 50% tissue culture infectious dose (TCID_50_) assay. Briefly, HEK293 cells were plated into 96-well tissue culture plates at 5×10^3^ cells per well, and then serial dilutions of viral stock were added directly to the cells. Cells were incubated for 14 days, and relative cell density was determined using a crystal violet staining assay. Cell culture medium was removed and surviving cells were then fixed and stained with 2% (w/v) crystal violet (Sigma-Aldrich) in 70% ethanol for 3 hours at room temperature. The plates were extensively washed, air-dried, and optical density was measured at 570 nm using a V Max plate reader (Molecular Devices Corporation, Sunnyvale, CA). The number of wells with observable cytopathic effect per each row was determined. The viral titer was calculated by the Karber equation: T  =  10^1+D(S-0.5)^ × V^−1^, where T is infectious titer in TCID_50_ ml^−1^, D is the log_10_ of the dilution, S is the log_10_ for the initial dilution plus the sum of ratios, and V is the volume in ml of the diluted virus used for infection. Adenoviral vectors with the viral particle concentrations 1.1−2.9×10^12^ vp ml^−1^, 1.3−2.0×10^10^ TCID_50_ ml^−1^ and 1∶84-1∶92 TCID_50_ to vp ratios were used in presented study.

### Ethics statement

The Animal Studies Committee of Washington University in St. Louis approved all procedures under protocol numbers 20120029 and 20110035. During surgery for orthotopic tumor implantation, animal pain and suffering was minimized by using inhalational anesthesia and postoperative analgesia.

### Generation of composite mice

*Rag2^−/−^* (*Rag2^tm1.1Cgn^*) mice [13], in a mixed genetic background, were bred in house. Transgenic human coxsackie virus and adenovirus receptor (*hCAR*) mice on a mixed genetic background, likely C57Bl6/J and DBA [14], were obtained from Sven Pettersson. *ROSAR26R* (*Gt(ROSA)26Sor^tm1Sor^*) knock-in mice were obtained in house. *Rag2^−/−^* mice were serially intercrossed with *R26R* and *hCAR* transgenic mice to generate the composite mouse line, *hCAR/wt:R26R/R26R:Rag-2^−/−^*, subsequently denoted as *hCAR:Rag2^−/−^* mice. The warfarin liver detargeting experiments were performed using *wt/wt:R26R/R26R:Rag2^−/−^* littermates. The *R26R* conditional *LacZ* alleles were designed for Cre recombination experiments but were not used in this study.

### Creation of orthotopic and subcutaneous heterotopic tumors

Mice from 6 to 12 weeks of age were used in the present work. The 786-O human kidney cancer cell line was freshly obtained from ATCC and cultured in RPMI with 10% FBS with penicillin/streptomycin/amphotericin B. Xenograft tumors were established by injection of 5×10^6^ cells in 50 µl of RPMI media using aseptic technique. Kidney orthotopic tumors were established by left kidney subcapsular injection of 4×10^6^ 786-O cells in 40 µL of RPMI media. Carprofen, 5 mg/kg sc X 3 days, (Pfizer Animal Health, NY, NY) was used for postop analgesia. A total of 36 mice were both injected with 786-O cells and subsequently with Ad vectors (see below). As preliminary experiments revealed a 100% kidney orthograft “take” rate, bioluminescence imaging was not preformed in this study. Mice were sacrificed 4–6 weeks post 786-O injection when the subcutaneous xenograft tumors reached a diameter of 4 mm.

### Ad vector injections, host organ, and tumor harvest

Mice harboring established subcutaneous and kidney tumors were tail vein injected with 5.0×10^10^, 1.0×10^11^, or 1.5×10^11^ viral particles of Ad5ROBO4-EGFP or Ad5CMV-EGFP in 200 µl of saline. For warfarin experiments, mice were administrated warfarin (5 mg/kg) dissolved in peanut oil subcutaneously on day −3 and day −1 prior to vector injection. Organs and tissue were harvested from mice anesthetized with 2.5% 2, 2, 2-tribromoethanol (Avertin, Sigma-Aldrich, St. Louis, MO) 72 hours post vector injection for all experiments.

### Tissue harvest and immunofluorescent localization of reporter gene expression

Mice under Avertin anesthesia were perfused via the left ventricle with phosphate-buffered saline (PBS, pH 7.4), followed by 4% paraformaldehyde/PBS for whole body fixation. Mouse organs and tumors were collected, post-fixed in 4% paraformaldehyde for 2 hours at room temperature, cryopreserved in 30% sucrose for 16 hours at 4°C, and cryo-embedded in NEG50 (Thermo Fisher Scientific, Waltham, MA) over 2-methylbutane/liquid nitrogen. Sixteen-micrometer frozen sections were air-dried, washed in PBS, blocked with protein block (1% donkey serum in PBS containing 0.1% Triton X-100), and incubated with primary antibodies including: rat anti-endomucin, 1∶1,000, (#14-5851-81 eBioscience, San Diego, CA), Armenian hamster anti-CD31, 1∶1,000, (#MAB1398Z EMD-Millipore, Billerica, MA), and rabbit anti-GFP, 1∶400, (#A11122 Life Technologies, Carlsbad, CA). After PBS washes, the slides were incubated with corresponding Alexa Fluor 488 and Alexa Fluor 594, 1∶400, (Jackson ImmunoResearch Laboratories, West Grove, PA) conjugated secondary antibodies and counterstained for nuclei with SlowFade Gold Antifade mounting reagent with 4′,6-diamidino-2-phenylindole (DAPI) (Life Technologies). Fluorescence microscope images were collected using an FVII digital camera with Extended Focal Imaging (EFI) function (Olympus America, Center Valley, PA). To quantify the tissue section GFP fluorescence, the areas of GFP (+) cells and dual CD31/endomucin (+) blood vessels were measured and normalized by total tissue area per field. Imaging experiments were repeated 2–4 times on independent sets of vector-injected mice. Quantitative imaging experiments were based on the ratio of Ad5ROBO4 vector expression (green fluorescence emission from fluorophore tagged the anti-EGFP secondary antibody) divided by total vascular endothelial area (red fluorescence emission from the fluorophore tagged anti-CD31/endomucin secondary antibodies) using image analysis software (MicroSuite Biological Suite Version 5, Olympus), from 4–5 mice in each group.

### Cultured cell, tissue, and whole organ protein expression analysis by immunoblotting

Anesthetized mice were perfused via the left ventricle with cold phosphate-buffered saline (PBS, pH 7.4) containing 1 mM PMSF (Sigma-Aldrich), organ tissues and tumors were snap frozen in liquid nitrogen, and stored in the liquid nitrogen vapor phase. Frozen tissues were pulverized using a liquid nitrogen-chilled mortar and pestle (Cell Crusher, Thermo-Fisher), and the crushed powder lysed on ice in radioimmunoprecipitation assay buffer (RIPA; 20 mM Tris-HCl (pH 7.6), 0.15 M NaCl, 1% sodium deoxycholate, 1% NP40, 1 mM EDTA, 1 mM EGTA) supplemented with Protease Inhibitor Cocktail, 1∶10, Sigma-Aldrich) for 30 minutes. The late log-phase 786-O cells grown on a 10-centimeter tissue culture dish was washed once with cold PBS, and lysed on ice with 500 µl cold RIPA buffer supplemented with protease inhibitors (see above). Protein lysates were separated on polyacrylamide gels and transferred to polyvinylidene difluoride (PVDF) membranes. Protein loading in individual lanes was normalized first to β-tubulin and then VE-Cadherin. Membranes were blocked in Tris-buffered saline (TBS, pH 7.6) containing 0.5% Tween 20 (TBST) and 5% nonfat dry milk and incubated in 5% BSA in TBST, containing the following antibodies: rabbit polyclonal anti-ROBO4 (generous gift from Dean Li, University of Utah), chicken anti-EGFP, 1∶1,000, (#A10262 Life Technologies), goat anti-VE-Cadherin, 1∶400, (#A1002 R&D Systems, Minneapolis, MN), and polyclonal anti-β-tubulin, 1∶20,000, (Novus Biologicals, Littleton, CO) overnight. Membranes were washed three times with TBST and incubated in TBST containing 5% milk with the corresponding IgG-horseradish peroxidase conjugate, 1∶5,000, (Santa Cruz Biotechnology, Santa Cruz, CA) for 1 hour. After three TBST washes, peroxidase activity was revealed by enhanced chemiluminescence using ECL2 or SuperSignal West Femto Western Blotting Substrate (both from Thermo Scientific) and imaged using a Chemidoc XRS imaging system (Bio-Rad Laboratories, Hercules, CA). The immunoblotting was quantified by densitometry with Quantity One one-dimensional analysis software (Bio-Rad Laboratories). Immunoblotting was repeated 2–4 times using extracts from independent experiments from separate sets of mice. Representative blots and densitometry analysis were presented for each group of immunoblots in each experiment.

### Statistical analysis

Significance between groups in the differential fluorescent area experiments was determined using one-way ANOVA with Tukey's correction for multiple group comparisons (GraphPad Prism, San Diego, CA).

## Results

### Endogenous ROBO4 in renal cancer xenografts and orthotopic tumors

**Figure 1:**
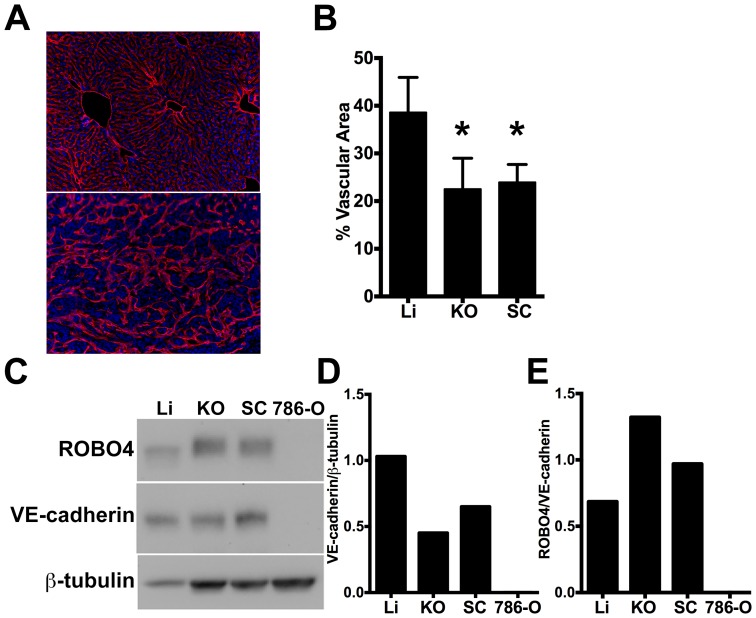
Endogenous ROBO4 upregulation despite lower vascular density in orthotopic and xenograft tumors. A. Immunofluorescence of the vascular endothelium in liver (upper panel) and 786-O human renal cell carcinoma (RCC) subcutaneous xenograft tumor (lower panel). B. Vascular area analysis of liver (Li), kidney orthotopic (KO) tumors, and subcutaneous (SC) xenograft tumors (n = 6 mice analyzed). C. Immunoblot of endogenous ROBO4 and the endothelial cell specific VE-cadherin from liver, kidney orthotopic and subcutaneous xenograft 786-O RCC tumors, and from the derivative 786-O cells grown in culture. D. Densitometry analysis of VE-cadherin/tubulin ratio from C mirrors the vascular area determination in B. E. Densitometry analysis of endogenous ROBO4 normalized to VE-cadherin expression reveals a 1.4- to 2-fold increase in SC and KO tumors compared to liver. C–E: Immunoblot and densitometry was repeated twice with two independent sets of protein extracts from two different tumor-bearing mice with essentially the same results. A. Magnification: 100X, Red: endomucin/CD31 antibody cocktail, Blue: DAPI. B. *p<0.05, one way ANOVA with Tukey's correction, mean ± SD.

**Figure 2:**
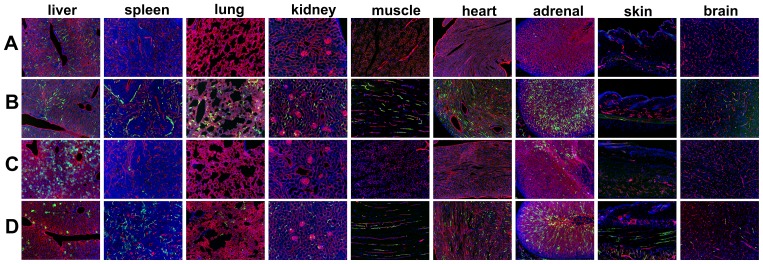
Adenoviral Type 5 (Ad5) vector expression in a host organ panel in tumor bearing immunodeficient *Rag2^−/−^*, and *hCAR:Rag2^−/−^* composite mice. Host organ EGFP reporter expression following intravenous injection of 1.0×10^11^ viral particles (vp) of either (A, B) Ad5ROBO4-EGFP (ROBO4) or (C, D) Ad5CMV-EGFP (CMV) vectors. A. Ad5ROBO4 vector expression in *Rag2^−/−^* mice is widespread but focal in liver endothelial cells (ECs), and rarely detectable in single splenic microvessels. All other organs are negative for vector reporter expression. B. Multiorgan, EC restricted vascular expression is evident in Ad5ROBO4-injected *hCAR:Rag2^−/−^* composite mice. C. Ad5CMV vector expression in *Rag2^−/−^* mice is detectable in liver hepatocytes, sporadic splenic (inflammatory) and adrenal cells. D. Ad5CMV vector expression in *hCAR:Rag2^−/−^* mice is complex, hepatocyte localized, but decreased in frequency compared to C in liver, mixed inflammatory and endothelial cell localized in spleen, and EC localized in all other organs including brain and skin. A and B: n = 5–6 mice, combined from 3–6 independent experiments, C and D: n = 3–4 mice combined from 3–4 independent experiments Magnification: 100X, Red: endomucin/CD31, Green: EGFP immunofluorescence, Blue: DAPI.

While investigators agree that ROBO4 expression is restricted to ECs and bone marrow hematopoietic stem cells [15]–[17], ROBO4 expression level in normal organs and particularly in tumors is controversial [18]–[22]. The 786-O human kidney cancer cell line was chosen for our proof of concept tumor endothelial cell (EC) expression engineering because these cells form hypervascular tumors in immunodeficient hosts due to constitutive, high level expression of hypoxia-inducible factor-2, and its downstream target vascular endothelial growth factor [23], [24]. To determine differential expression of ROBO4 in this kidney cancer model, and to provide a backdrop for our investigation of the human *ROBO4* enhancer/promoter fragment used in our Ad5ROBO4-EGFP vector, we tested ROBO4 protein levels in 786-O orthotopic (KO) and subcutaneous (SC) tumors compared to liver using a validated ROBO4 antibody (Figure 1) [25], [26]. Prior to immunoblotting, we noticed that the 786-O tumor vascular density appeared to be less than that of normal organs despite its hypervascularity in tissue sections stained with a CD31/endomucin cocktail (Figures 1A and 2). Indeed, image analysis revealed a 1.6- and a 1.7-fold lower vascular area density in KO and SC 786-O tumors, respectively, compared to liver (Figure 1B). Based on this vascular density analysis, we immunoblotted for the EC specific marker, VE-cadherin in our ROBO4 immunoblots and all subsequent Westerns to normalize for this differential tumor/host organ vascular density. Consistent with the tissue immunofluorescence, densitometric analysis of VE-cadherin immunoblotting revealed a 2.3- and 1.6-fold decrease in KO and SC tumor versus liver expression (Figures 1C and 1D). Liver was selected as our control normal organ because of its avid Ad vector uptake [27], [28]. To further test for ROBO4 endothelial specificity, 786-O cells grown in culture were lysed and run on the same immunoblots as livers and tumors obtained from the mice. Compared to prior reports suggesting robust ROBO4 upregulation in tumor angiogenesis [12], [17], [18], endogenous ROBO4 was very modestly upregulated 2.0-fold in KO, and 1.4-fold in SC 786-O tumors, compared to liver (Figure 1E).

### Endothelial specificity of the Ad5ROBO4 vector following systemic administration

While the 3 kb enhancer promoter fragment of human *ROBO4* enhancer/promoter had been previously validated for endothelial expression in single copy and endogenous locus transgenic knock-in mice [12], [29], there was no guarantee of its similar specificity when used in the context of an Ad vector. Therefore to test expression localization, we compared our Ad5ROBO4 versus Ad5CMV vectors in a nine organ panel harvested from tumor bearing immunodeficient *Rag2^−/−^* mice (negative littermate mice derived from *hCAR:Rag2^−/−^* × *Rag2^−/−^* matings, Materials and Methods) (Figures 2A and 2C). Ad5ROBO4-mediated reporter gene expression was exclusively EC localized predominantly in the liver, with sporadically detectable rare EC expression in spleen and heart (Figure 2A). In stark contrast, Ad5CMV-mediated EGFP expression was predominantly detectable in liver hepatocytes, and occasional reticuloendothelial system (RES) cells in spleen and adrenal gland (Figure 2C). Large format images highlighted the potent cell type specific transcriptional retargeting from hepatocytes to liver ECs mediated by the 3 kb *ROBO4* enhancer/promoter (Figure S1).

### Enhanced but promiscuous endothelial Ad vector expression mediated by ubiquitous hCAR transgene expression

As undetectable host organ Ad5ROBO4 vector expression was likely due to extremely low levels of EC CAR protein, we intercrossed *Rag2^−/−^* with *hCAR* transgenic mice to test the maximal EC expression capacity of our Ad5ROBO4 vector. We performed comparative experiments by intravenously injecting 1.0×10^11^ vp of either Ad5ROBO4-EGFP or Ad5CMV-EGFP vectors into *hCAR:Rag2^−/−^* tumor bearing composite mice, but for this analysis focused on host organs (Figures 2B and 2D). Endothelial expression was markedly increased in all organs except for liver and skin in Ad5ROBO4 injected mice (Figure 2B). The Ad5CMV vector expression pattern in composite mice was surprisingly complex (Figure 2D). Frequent EC expression was detected in kidney glomeruli and peritubular vessels, muscle, myocardium, adrenal and skin, with focal expression in brain vessels (Figure 2D). In sharp contrast, liver Ad5CMV-mediated EGFP expression remained restricted to hepatocytes, while splenic expression was a composite of vessel ECs and interfollicular RES cells (Figure S1 and Figure 2D). Of interest, and consistent with an intravenous injection “first pass” phenomenon, appreciable hepatocyte detargeting was produced by ubiquitous hCAR transgene expression (Figure S1).

### Ad5ROBO4 transcriptionally targets tumor endothelial cells

**Figure 3:**
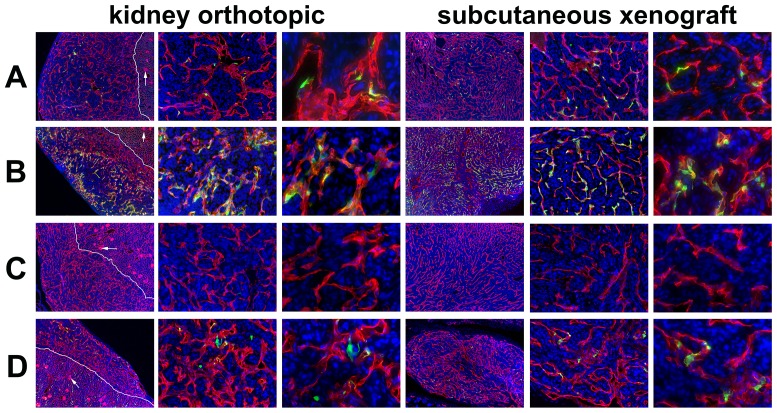
Vascular restricted ROBO4-directed reporter expression in kidney orthotopic and subcutaneous xenograft tumors. The tumors were harvested from the same Ad vector injected mice whose host organs were depicted in Figure 2. A. Ad5ROBO4 vector expression in *Rag2^−/−^* mice is sporadic yet easily detectable in vascular ECs within tumors in either orthotopic or subcutaneous microenvironments. B. The *hCAR* transgene markedly increases Ad5ROBO4 vector expression throughout both the orthotopic and subcutaneous tumors following injection into *hCAR:Rag2^−/−^* mice. C. Ad5CMV expression in *Rag2^−/−^* mice is undetectable in either orthotopic or subcutaneous tumors. D. Isolated intratumoral endothelial cell reporter expression in *hCAR:Rag2^−/−^* mice. White line: kidney-tumor boundary; Arrows: glomerular tufts. Magnifications: 40X, first and fourth columns, and 200X, second and fifth columns, 400X, third and sixth columns. Red: endomucin/CD31, Green: EGFP immunofluorescence, Blue: DAPI.

After elucidation of EC expression patterns of the Ad5ROBO4 and Ad5CMV vectors in host organs either without (Figures 2A and 2C) or with (Figures 2B and 2D) the hCAR transgene, we next tested for differential vector expression in 786-O tumors (Figure 3). In contrast to our nine-organ host panel wherein Ad5ROBO4 vector expression was essentially restricted to liver ECs, both KO and SC tumors evidenced easily detectable, though sporadic, Ad5ROBO4 EC EGFP immunofluorescence in *Rag2^−/−^* mice (Figure 3A). The addition of the hCAR transgene produced markedly increased intratumoral Ad5ROBO4 vessel expression (Figure 3B). To test for tumoral Ad5ROBO4 mediated EC cell type specificity, we co-stained tumor sections with antibodies cognate for pericyte, desmin, PDGFRβ, and NG2, or inflammatory cell, CD45, antigens (Figure S2). As a subset of intratumoral vascular pericytes remained intimately associated with capillary ECs, a fraction of these cells labeled with the red-emitting fluorochrome appeared to partially co-localize with the green EGFP immunofluorescent signal. However, closer examination revealed distinct segmentation of vector transgene expression to ECs rather than overlying pericytes (Figure S2). These data supported the EC specificity of the Ad5ROBO4 vector in the intratumoral microenvironment. In contrast to Ad5ROBO4, Ad5CMV-mediated EGFP expression was undetectable in KO and SC tumors grown in *Rag2^−/−^* mice (Figure 3C). The presence of the hCAR transgene only elevated intratumoral Ad5CMV-mediated EGFP expression to a level approaching, but still lower than, that of Ad5ROBO4 in hCAR negative mice (Figure 3D versus 3B). Moreover, only half of the sporadically dispersed intratumoral EGFP positive cells in Ad5CMV vector injected *hCAR:Rag2^−/−^* mice were co-localized within ECs, while the other half were perivascular stromal cells (Figure 3D, third and sixth columns, 400X magnification).

**Figure 4:**
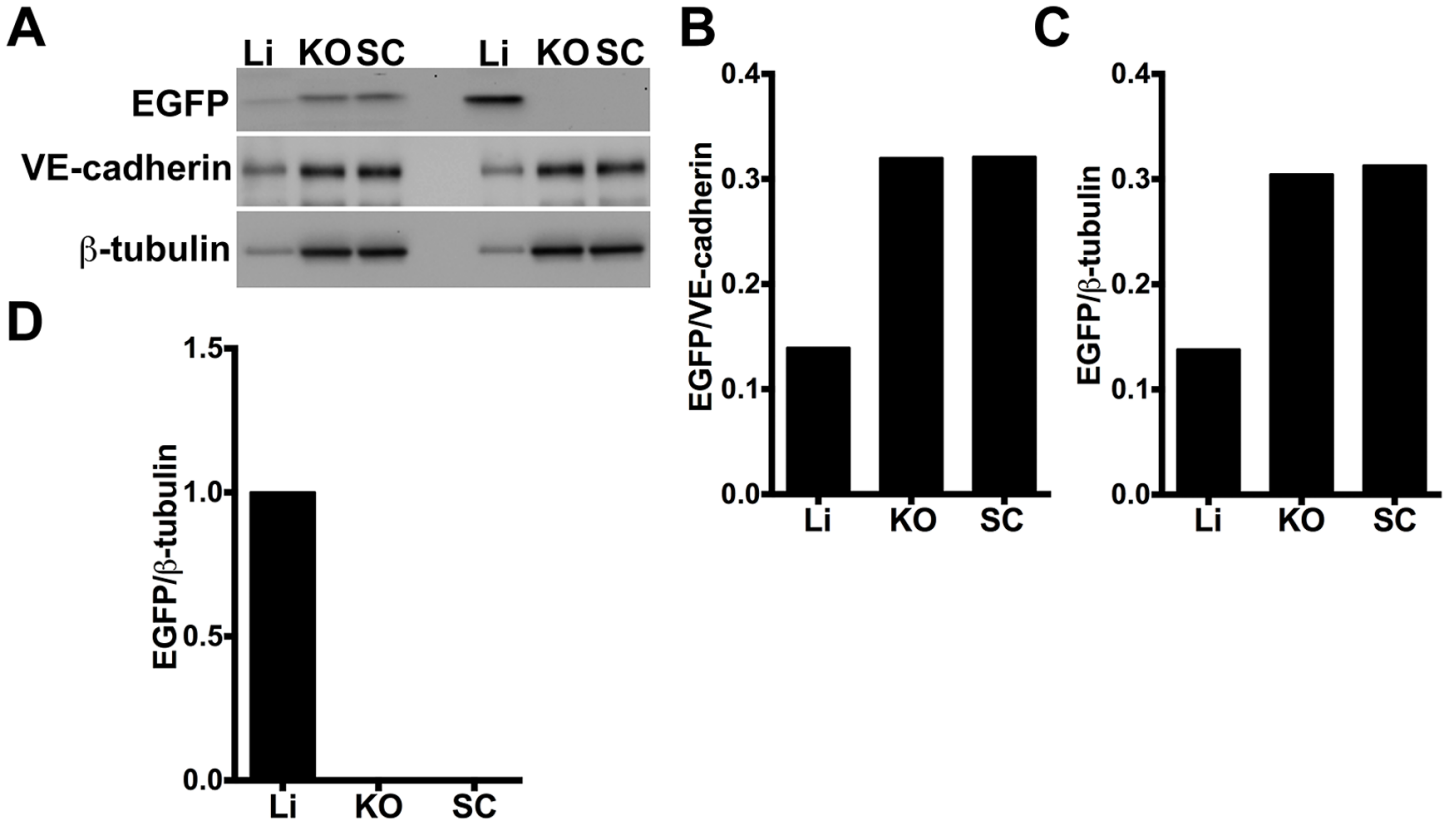
Semiquantitative immunoblotting reveals differential Ad5ROBO4 reporter expression in tumor compared to liver. A. Immunoblot of EGFP, VE-cadherin, and β-tubulin loading controls in tissue protein extracts from *hCAR:Rag2^−/−^* mice injected with either Ad5ROBO4, left three lanes, or Ad5CMV, right three lanes. B. and C. Densitometry analysis of Ad5ROBO4 vector EGFP expression normalized to either VE-cadherin or β-tubulin. D. Densitometry of AdCMV vector EGFP expression. As AdCMV expression was hepatocyte specific, this blot was only normalized to β-tubulin. A–D: Representative immunoblots from n = 4 mice injected with either Ad5ROBO 4 or Ad5CMV vectors. Li: liver, KO: kidney orthotopic tumor, SC: subcutaneous tumor.

To semiquantitatively test for differential Ad vector-mediated host organ versus tumor expression, we immunoblotted extracts from both KO and SC tumors as well as liver, in *hCAR:Rag2^−/−^* mice injected with 5.0×10^10^ vp of either the Ad5ROBO4 (n = 4 mice) or Ad5CMV (n = 4 mice) vectors, and probed for EGFP protein expression normalized to either VE-cadherin or β-tubulin (Figures 4A–D). Four independent experiments yielded consistent results, and one representative data set of the four is presented in Figure 4A. The near total absence of Ad5CMV-regulated tumor immunofluorescent expression (Figure 3D) was validated as neither the orthotopic nor xenograft immunoblots contained detectable EGFP protein (Figures 4A and 4D). In contrast, KO and SC tumor extracts from Ad5ROBO4 injected mice contained easily detectable EGFP expression that was 2.1- to 2.3-fold elevated compared to liver when normalized to either β-tubulin or VE-cadherin respectively (Figures 4A, 4B, and 4C).

### Ad5ROBO4 vector transcriptionally targets metastatic tumor endothelial cells

During tissue immunofluorescence analysis we serendipitously detected intra-ovarian and peritoneal metastases in an Ad5ROBO4 injected (1.5×10^11^ vp) *hCAR:Rag2^−/−^* mouse bearing an orthotopic tumor (Figure S3A–D). Nearly all of the microvessels within the intra-ovarian and peritoneal metastases expressed EGFP. In contrast, there was almost no expression within stromal ECs within the metastasis-bearing ovary except for perifollicular microvessels (Figure S3A–3C). Nor was expression detectable in ECs within the fallopian tube abutting the peritoneal metastasis (Figure S3D).

### Pharmacological liver detargeting increases the tumor EC expression bias of the Ad5ROBO4 vector

**Figure 5:**
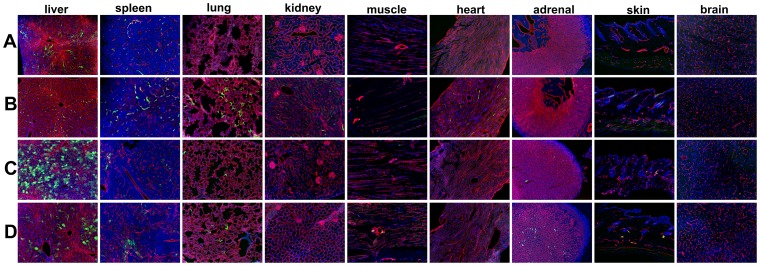
Warfarin pretreatment “detargets” the liver without producing multiorgan Ad5ROBO4 vector expression. A. Ad5ROBO4 injection into vehicle-treated *Rag2^−/−^* mice produced vascular EC expression predominantly in liver and spleen. B. Warfarin pretreatment, 5 mg/kg, on day −3 and −1 prior to Ad5ROBO4 injection vector injection increased the frequency of splenic and lung EC expression and produced sporadic, infrequent expression in kidney and heart. Liver expression was present but diminished. C. AdCMV injection into vehicle treated mice predominantly produced hepatocyte expression with focal RES cell splenic and scattered lung expression. D. Warfarin pretreatment prior to AdCMV injection markedly decreased the frequency of hepatocyte EGFP expression, while increasing sporadic splenic, and lung expression, inducing focal adrenal cellular and rare muscle and skin EC expression. A, B and S5: Representative images from n = 5 mice from 2 independent experiments, C and D: n = 3 mice from 3 independent experiments. 1×10^11^ vp were injected in each group. Magnification 100X; Red: endomucin/CD31, Green: EGFP immunofluorescence, Blue: DAPI.

As real world clinical applications demand sufficient target cell vector payload expression in the context of low hCAR expressing ECs, we manipulated *Rag2^−/−^* host mice attempting to decrease liver vector uptake and conversely increase tumor viral particle delivery without the necessity of the *hCAR* transgene. For simplicity and across the board comparative analyses we chose warfarin administration, because the predominant mechanism for Ad vector liver sequestration is mediated by coagulation Factor X-viral capsid hexon binding [30], [31]. However, it is important to keep in mind that levels of vector liver detargeting similar to warfarin can be achieved using vector capsid mutation(s) [31]–[33]. First we tested liver detargeting efficiency in our *Rag2^−/−^* mice. Warfarin, 5 mg/kg, pretreatment on day −3 and −1 before injection of 1.0×10^11^ vp AdCMV-EGFP, revealed a marked diminution of hepatocyte reporter expression (Figures 5C, 5D and S4). Compared to the multiorgan EC expression mediated by the *hCAR* transgene (Figures 2B and 2D), warfarin failed to achieve appreciable multiorgan EC expression in Ad5CMV injected mice (Figures 5C and 5D) except for clusters of EGFP positive RES cells in the spleen, sporadic EC expression in the lung and adrenal gland, and only 1–4 individual positive ECs in muscle and skin. Brain lacked Ad5CMV EC vector expression. Encouragingly, warfarin treatment prior to Ad5ROBO4 vector injection produced a similar paucity of host organ EC expression other than in liver and spleen, and very focal areas in the lung (Figures 5A, 5B and S5).

### Ad5ROBO4 EC targeting is maintained and differentially enhanced in both orthotopic and xenograft tumors compared to host organs following warfarin-liver detargeting

**Figure 6:**
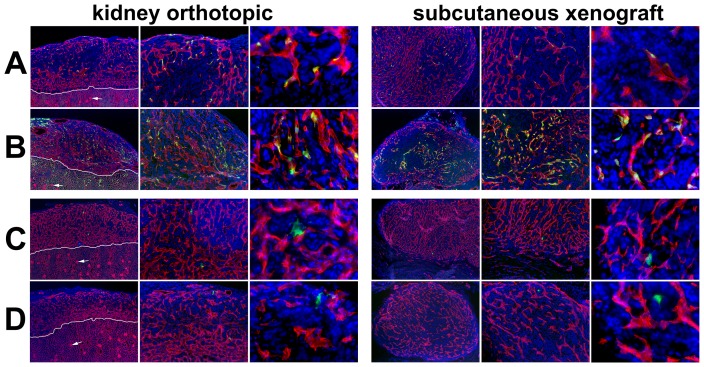
Warfarin liver detargeting enhances tumor neovascular endothelial cell reporter expression of the Ad5ROBO4 vector. A. Ad5ROBO4 produced easily detectable scattered tumor endothelial cell EGFP immunofluorescence in both kidney orthotopic and subcutaneous 786-O tumors in vehicle-treated *Rag2^−/−^* mice. B. Warfarin pretreatment markedly enhanced the multiplicity of tumor endothelial cell reporter gene expression within both orthotopic and subcutaneous tumors in Ad5ROBO-injected mice. C. Ad5CMV injection failed to produce detectable tumor EC expression in vehicle-treated, or D. warfarin-treated mice. A–D: tumors from the same mice as in Figure 5. Magnifications: 40X first and fourth columns, 100X second and fifth columns, and 400X third and sixth columns. Red: endomucin/CD31, Green: EGFP immunofluorescence, Blue: DAPI.

As warfarin-mediated liver detargeting effectively eliminated Ad5ROBO4-mediated EC expression in most host organs examined (Figure 5B and S5), our next question was whether EC expression in the tumors of mice whose host organs were examined in Figure 5, would be similarly diminished (Figure 6). Similar to data reported in Figure 2, scattered EC vector expression was detectable in both KO and SC tumors of vehicle (peanut oil) treated, Ad5ROBO4-injected mice (Figure 6A). Warfarin pretreatment produced a heterogeneous, but high level, regional increase in intratumoral EC vector expression (Figure 6B). In contrast, Ad5CMV expression was undetectable in intratumoral vessels either without or with warfarin pretreatment (Figure 6C and 6D). To quantify warfarin's effects on Ad5ROBO4-mediated EC expression in KO and SC tumors versus host organs, we measured the area of EC co-localized reporter versus total EC immunofluorescent areas in tissue sections from Ad5ROBO4-injected vehicle (n = 4) or warfarin-injected (n = 5) mice (Figure S5). Warfarin produced an eight-fold increase in EGFP-EC co-localization in orthografts and a six-fold increase in xenografts (p<0.05 warfarin compared to vehicle, Figure S5). Five of seven host organs evidenced minimal areas positive for Ad5ROBO4 expression, albeit with single outlier mice in each organ. Warfarin produced a 1.7-fold decrease in EGFP-EC co-localization in liver and a 2.6-fold increase in co-localization in the spleen (p<0.05 compared to vehicle, Figure S5).

**Figure 7:**
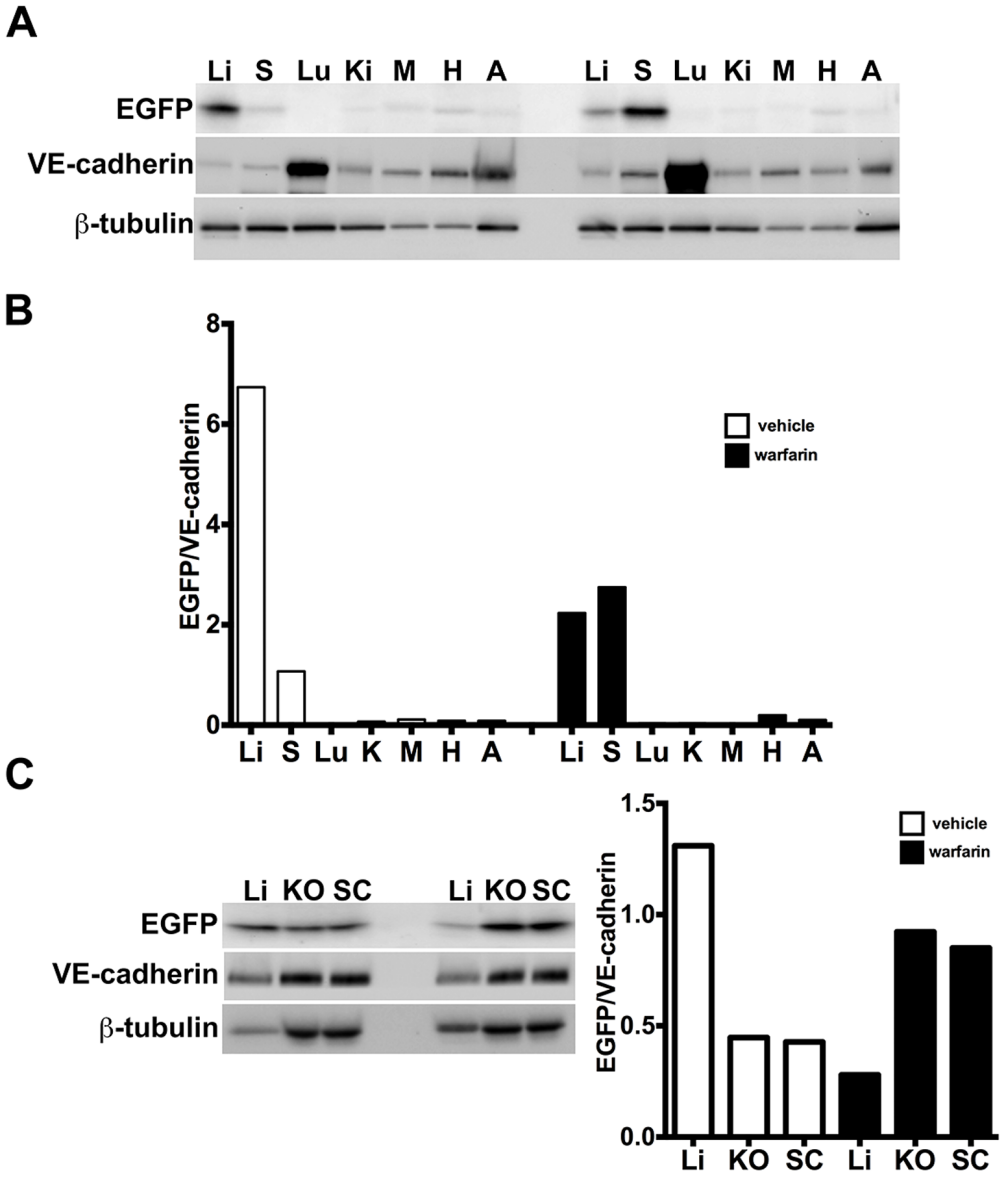
Warfarin pretreatment enhances tumor Ad5ROBO4 vector expression. A. Multiorgan immunoblot of vehicle (left lanes) or warfarin (right lanes) pretreated *Rag2^−/−^* mice injected with 1.0×10^11^ vp of Ad5ROBO4. B. Densitometry of A revealed that vehicle pretreatment was associated with robust liver, detectable splenic, and trace to undetectable expression in all other sampled organs. Warfarin pretreatment produced a 2.5-fold increased splenic and a 3-fold decreased liver expression while all other organs still evidenced trace to undetectable expression. C. Immunoblot and densitometry of liver and tumor EGFP, VE-cadherin, and β-tubulin expression in vehicle (left lanes) or warfarin (right lanes) from the same pretreated, Ad5ROBO4-injected mice as in A and B. EGFP densitometry normalized to VE-cadherin, revealed a 4.7-fold decrease in liver and 2-fold increase in increase KO and SC tumor expression produced by warfarin pretreatment. A–C: representative immunoblots from n = 2 mice from 2 independent experiments.

To quantitatively test for warfarin mediated shifts of host organ versus tumor vector expression we immunoblotted tissue extracts, probing for EGFP, VE-cadherin, and the β-tubulin loading control (Figures 7A–7C). As the ultimate question was the warfarin-mediated differential tumor EC versus host organ expression, we focused our experiments exclusively on mice injected with the Ad5ROBO4 vector. Host organ whole tissue extract immunoblotting of vehicle treated mice substantiated the markedly predominant liver vector expression (Figure 7A, left seven lanes and 7B, white bars). Warfarin pretreatment produced a 2.5-fold increase in spleen and a 3-fold decrease in liver vector expression (Figure 7A, right seven lanes and 7B, black bars). Following this determination of host organ expression distribution, the effect of warfarin on tumor versus liver expression was tested in independent immunoblotting of KO, SC, and liver whole tissue extracts (Figure 7C). Warfarin pretreatment produced a 2.1-fold increase in KO, a 2.0-fold increase in SC, and a 4.7-fold decrease in liver vector expression normalized to VE-cadherin compared to vehicle (Figure 7C, left three gel lanes vehicle, right three lanes warfarin). The fold-increase in tumor Ad5ROBO4 expression mediated by warfarin-liver detargeting was closely comparable to that produced by ubiquitous *hCAR* transgene expression, and reinforces the conclusion that the Ad5ROBO4 vector is preferentially, though not exclusively, expressed in tumor versus host organ endothelium.

## Discussion

The tumor gene therapy field is challenged by several key issues; target cell vector transduction, hepatic toxicity due to viral gene expression, and innate and adaptive host vector immune response [27], [34]. ECs are enticing targets for vector-mediated genetic manipulation as they are the first cells exposed to intravenously injected particles. Moreover, as tumor microvessels are conduits distributed throughout tumors, particularly in hypervascular tumors such as renal cancer, EC targeting in combination with systemic vector injection can overcome intratumoral vector distribution obstacles inherent in local injection. Here we took our first steps towards EC targeting using a vector transcriptional approach. The use of a previously characterized 3 kb enhancer/promoter of the human *ROBO4* gene [12] produced vascular endothelial localized gene expression in mice injected with the Ad5ROBO4 vector. This Ad5ROBO4 vector also predominantly targeted the endothelium within renal cancer xenografts, subcapsular orthotopic and metastatic tumors. In addition, we discovered that experimental renal cancers evidenced a remarkable pharmacological induction of tumor EC vector expression compared to the concomitant decrease in the target organ most at risk for adenoviral toxicity, the liver.

Gene therapy approaches to the vascular endothelium have utilized several approaches. Vector-host cell transduction was manipulated to produce tumor EC targeting [35], [36]. Adenoviral and adeno-associated vectors were engineered for capsid display of peptides identified on tumor-activated endothelium, or bispecific antibodies cognate for integrins, selectins, or vessel luminal cell surface receptors [37]–[39]. Vector pseudotyping using fiber-knobs from serotypes other than adenovirus type 5, other animal host species, or fiber replacements, either from other viruses or virus-synthetic chimeric fibers, also achieved EC tropism [38], [40]. Some studies focused on the dual goals of liver sequestration inhibition, and hCAR de-targeting concomitant with tumor EC transductional targeting [37]. EC specific transcriptional targeting efforts have used vectors containing enhancer/promoter elements that are induced by intratumoral growth factor production or hypoxia [11], [41], [42]. EC production of conditionally replicative adenoviral vectors have been constructed engaging the dual strategies of tumor angiogenic factor induced EC proliferation, and DNA enhancer/promoters activated by intratumoral hypoxia [43]–[45]. Most recently, a vector designed and preclinically tested for tumor EC expression has advanced to a Phase I trial [46]. Despite this progress, detailed comparative analysis of preclinical studies using EC-targeted vectors has been challenging. Some studies were solely performed in cultured ECs [39], [47]. Bridging studies tested in vitro transduced ECs in mixed tumor-EC injections [48]. Other approaches used direct injection of vascular-targeted vectors into tumors [49]. These experimental strategies failed to address the crucial challenge of tumor vessel delivery following systemic administration that is the preclinical translational lynchpin. Prior work engaging systemic vector delivery predominantly used enzymatic luciferase assays of whole tissue that were not linearly quantitative and also failed to co-localize vector expression within ECs [44]. Studies documenting co-localization frequently presented “coned down” high magnification views of single vessels but failed to evaluate tumor-wide vascular distribution [37], [50], [51]. Our efforts focused on evaluation of vector reporter gene expression using a combination of wide field low power, intermediate, and high power microscopic magnification bolstered by quantitative immunoblotting. This experimental design allowed us to uncover definitive evidence for vascular EC vector co-localization within primary and metastatic cancers, and enabled us to screen multiple host organs to determine the degree of vector tumor EC specificity.

We focused our first-line study on endothelial transcriptional targeting of an Ad5 vector using a human *ROBO4* enhancer/promoter as previous work suggested that the endogenous gene was uniquely expressed in vascular ECs [17]. However subsequent studies in mice additionally detected endogenous *Robo4* expression in lymphatic endothelium, hematopoietic stem cells, and in neocortex neurons during embryonic development [15], [52], [53]. While core ROBO4 functions including EC migration, vascular permeability, and angiogenesis modulation have been delineated [54], the data vary according to species and context, and in some cases are contradictory. ROBO4 is necessary for angiogenesis zebrafish [55], but dispensable during normal mammalian development [25]. ROBO4 has been reported to either inhibit, [26], stimulate [56], or repulse [57] EC migration. Contextual modulation of EC biology by ROBO4 was particularly highlighted in the murine breast, wherein *Robo4* loss of function failed to affect the quiescent mammary gland, but negatively regulated pregnancy-associated, VEGF-mediated angiogenesis [21]. At the molecular level, ROBO4 was shown to bind paxillin ultimately leading to inhibition of Rac activation and lamellipodial formation via GIT1-GAP Arf6 GTPase inactivation [58]. Most of the ROBO4 functions were delineated using Slit proteins as presumptive ligands [21], [25]. However Biacore analysis of recombinant proteins failed to detect ROBO4-SLIT binding and instead, definitively demonstrated the UNC5B receptor as the ROBO4 binding partner [57], [59].

Similar to function, it has been challenging to discern a literature consensus on differential ROBO4 expression in tumor versus host ECs. ROBO4 has been suggested to be a tumor EC specific marker with minimal to undetectable expression in host ECs [17], [18], [20]. In contrast, studies using the 3 kb *ROBO4* enhancer/promoter engineered into our Ad vector, demonstrated multiorgan EC expression [12], [29]. However, as these were enzymatic LacZ assays, low-level EC expression would be amplified. Whole tumor ROBO4 expression assays revealed either induction [22] or downregulation [19]. Our immunoblotting analysis was consistent with both overexpression of endogenous ROBO4 in hypervascular 786-O renal KO and SC cancers, but also detectable host organ expression. However, our studies using *hCAR* nontransgenic mice clearly revealed differential Ad5ROBO4 expression in tumor versus most host organs except for spleen when liver vector sequestration was inhibited by warfarin. In the original studies of this 3 kb enhancer/promoter fragment ETS family and Sp1 transcription factors were necessary for the *ROBO4* enhancer/promoter fragment activity [12], [29]. These transcription factors may also mediate our Ad5ROBO4 differential tumor versus host organ vector expression [60].

As ECs have negligible CAR levels we first chose the *hCAR* transgenic mouse, to isolate transcription as a single experimental variable for our proof of concept experiments [14]. These mice ubiquitously express a truncated receptor lacking the cytoplasmic domain necessary for signaling, but retain Ad5 binding. In the original paper describing these mice, EC reporter gene expression was demonstrated in a limited number of tissues, brain and lung, using β-galactosidase [14]. Our multiorgan co-immunofluorescence analysis revealed promiscuous EC expression of Ad5CMV in multiple organs at levels comparable to Ad5ROBO4 particularly following high titer intravenous administration. The endothelial *CMV* promoter transcriptional activation was likely due to several elements including ETS, AP1, and NFkB sites [61]. The striking lack of hepatocyte vector reporter gene expression in Ad5ROBO4 injected mice, and markedly reduced expression in Ad5CMV vector injected *hCAR* transgenic mice, supported the concept that transcriptional or transductional retargeting can ameliorate the liver toxicity of Ad5 vectors even with no pharmacological or viral capsid alterations for hepatic sequestration diminution [31]. However, the promiscuous vascular EC expression of the Ad5CMV vector revealed the limited utility of *hCAR* transgenic mice as testing models for tumor selective endothelial expression. Despite this confounding host organ challenge, the striking differential Ad5ROBO4 versus Ad5CMV EC vector expression in tumors grown in *hCAR* transgenic/immunodeficient mice reinforced the notion of the Ad5ROBO4 vector expression bias in tumor ECs. The paucity of intratumoral EC expression in Ad5CMV injected mice was perplexing, but suggestive of the fact that the tumor microenvironment failed to upregulate transcription factors in the appropriate context to activate the *CMV* promoter.

Our most impressive finding was six to eight-fold induction of Ad5ROBO4 vector EC expression in tumors following factor X-mediated liver detargeting in *Rag2^−/−^* mice lacking the *hCAR* transgene. The only host organ whose EC expression increased followed warfarin was the spleen, but here the 2.6-fold induction was still lower than that of tumor. As endothelial CAR levels are extremely low, our data also suggested utilization of CAR independent vector transduction pathways in tumor and possibly splenic ECs, one candidate being α_v_/β_5_ integrin [62], [63]. As this integrin is differentially upregulated in tumor ECs [64], its potential facilitation of vector-tumor EC transduction could contribute to the differential expression bias of the Ad5ROBO4 vector in warfarin-liver detargeted mice.

Despite our encouraging data, we realize the need for further vector optimization to achieve both greater selective tumor EC expression specificity, and anticoagulant independent liver detargeting. It is likely that no enhancer/promoter will mediate tight expression restriction confined to tumor compared to host ECs. Even promising agents engineered for hypoxic induction likely mediate expression within organ regions with physiological hypoxia such as the kidney medulla and papilla, the centrilobular regions of the liver parenchyma, and the subendocardial zone of the heart. Thus, additional genetic engineering of this and likely other vectors with EC targeting capability will likely be necessary to enhance tumor versus host organ selectivity. Two candidate genetic manipulations include 3′ DNA sequences cognate for microRNAs downregulated in tumor versus host ECs [65], [66], or 5′ DNA elements whose secondary structure resolution requires the intracellular signaling milieu of growth factor activation for efficient vector transgene mRNA translation [67]. Genetic manipulation of the vector capsid will also be added to our EC-targeted vectors [32]–[34] to obviate the need for warfarin that is an obvious obstacle for clinical translation. However, it is most important to consider the goals of Ad vector tumor EC targeting. In the past, angiogenesis inhibition coupled with intratumoral microvascular ablation was paramount. Increasingly tumor vascular biologists are focusing on the flow-independent tumor growth promoting and perivascular trophic functions of ECs [9]. In this context, absolute tumor EC specificity might not be essential because the Ad vector mediated EC expression that is targeted to the perivascular tumor microenvironment may have minimal biological effect even though its present in normal organs such as the spleen. That said, enhancement of Ad5ROBO4 vector expression in tumor endothelium using liver detargeting opens the door for studies in a wide gamut of preclinical models to gain the most insight for optimal translational design of tumor EC-targeted vectors ultimately suitable for clinical trials.

## Supporting Information

Figure S1 **Large format views of Ad5ROBO4 endothelial specificity and liver detargeting mediated by ubiquitous hCAR expression in transgenic mice.** Magnification: 100 X. Red: endomucin/CD31 cocktail, Green: EGFP immunofluorescence, Blue: DAPI.
(TIF)

Figure S2 **Tumor Ad5ROBO4-EGFP expression is endothelial cell restricted.** 786-O kidney orthotopic (KO) and subcutaneous (SC) xenograft expression of the pericyte and stromal fibroblast markers desmin, PDGFRβ, and neural glial antigen-2 (NG2) is contained within a tissue compartment distinct from EGFP expressing cells. Similarly CD45 cells lack EGFP expression. Magnification: 200X. Red: each stromal protein; Green: EGFP immunofluorescence; Blue: DAPI.
(TIF)

Figure S3 **Ad5ROBO4 transcriptionally targets metastatic endothelium.** A–D. Intra-, and peri-ovarian “Krukenberg” renal carcinoma metastases from subcapsular 786-O orthografts in *hCAR:Rag2^−/−^* mice injected with 1.5×10^11^ vp display extensive and intense microvessel EGFP immunofluorescence. A. and B. Nearly all vessels within the two ovarian micrometastases (arrowheads) express the Ad5ROBO4 vector, whereas EGFP immunofluorescence is only detected in circumferential microvessels immediately adjacent to host ovarian follicles (asterisks), but not in stromal microvessels. C. Higher magnification view of one of the metastases revealing near ubiquitous intratumoral Ad5ROBO4 vector vascular expression. D. Near ubiquitous Ad5ROBO4 vascular expression is also evident in microvessels within a peritoneal metastasis adherent to the adjacent host fallopian tube (asterisks) whose vessels are negative for vector expression. Magnification: A and B 40X, C 200X, D 100X. A, C and D: Red: endomucin/CD31 cocktail, Green: EGFP immunofluorescence, Blue: DAPI. B: EGFP immunohistochemistry (brown) and hematoxylin counterstain.
(TIF)

Figure S4 **Large format view of warfarin mediated liver detargeting.** A. Vehicle (peanut oil) pretreated *Rag2^−/−^* mice injected with 1.0×10^11^ vp of Ad5CMV evidence EGFP expression localized to liver hepatocytes. B. Warfarin pretreatment markedly decreased the frequency of positive hepatocyte expression of the Ad5CMV vector, while producing sporadic expression in reticuloendothelial system and rare endothelial cells. Magnification: 100X. Red: endomucin/CD31 cocktail, Green: EGFP immunofluorescence, Blue: DAPI.
(TIF)

Figure S5 **Warfarin liver detargeting markedly increases intratumoral and splenic EGFP positive vascular areas without significant host organ expression.** Ratios of tissue section areas positive for EGFP colocalized with CD31/endomucin immunofluorescence over total CD31/endomucin immunofluorescence determined using image analysis software. In vehicle treated mice (n = 4), liver, spleen, kidney orthotopic and subcutaneous tumors are the only vascular beds with an appreciable extent of Ad5ROBO4 endothelial cell expression. Warfarin (n = 5 mice), mediated a marked enhancement of the extent of vector expressing tumoral vascular areas with a decrease in liver vascular area vector expression. Splenic vector positive area also appreciably increased with barely detectable induction in all other host organs except for a single outlier mouse. Blue dots: mean of four 100X fields for each vehicle-treated mouse, Red dots: mean of four 100X fields for each warfarin-treated mouse. *p<0.05 one-way ANOVA with Tukey's multigroup correction comparing vehicle and warfarin.
(TIF)
