# Supplementary figures and images for: Transcriptional Targeting of Primary and Metastatic Tumor Neovasculature by an Adenoviral Type 5 Roundabout4 Vector in Mice
Source: PLoS One. 2013 Dec 23;8(12):e83933. doi: 10.1371/journal.pone.0083933 (PMC3871592; doi:10.1371/journal.pone.0083933)

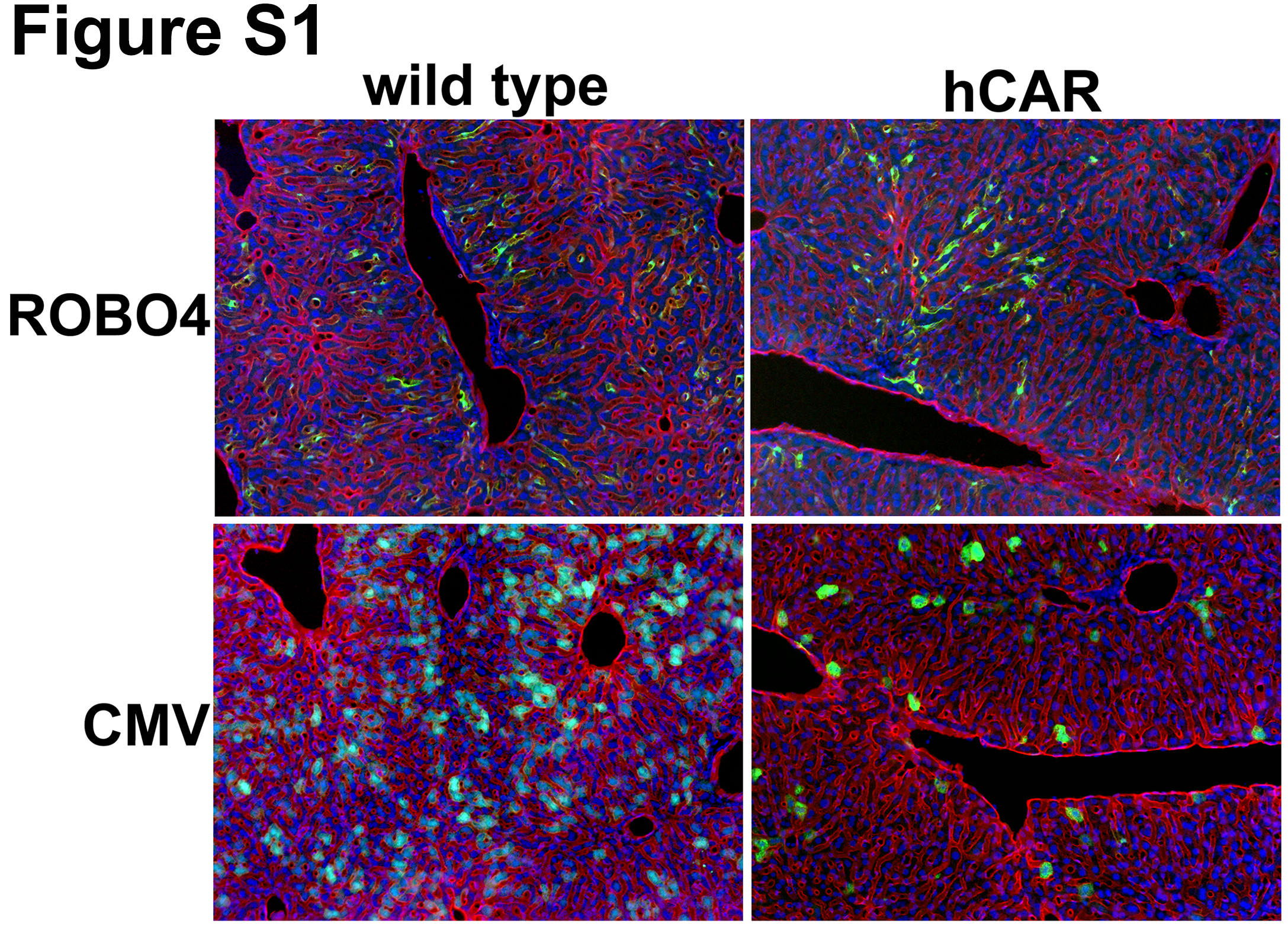

Supplement: Figure S1 — Large format views of Ad5ROBO4 endothelial specificity and liver detargeting mediated by ubiquitous hCAR expression in transgenic mice. Magnification: 100 X. Red: endomucin/CD31 cocktail, Green: EGFP immunofluorescence, Blue: DAPI. (TIF) [file pone.0083933.s001.tif]

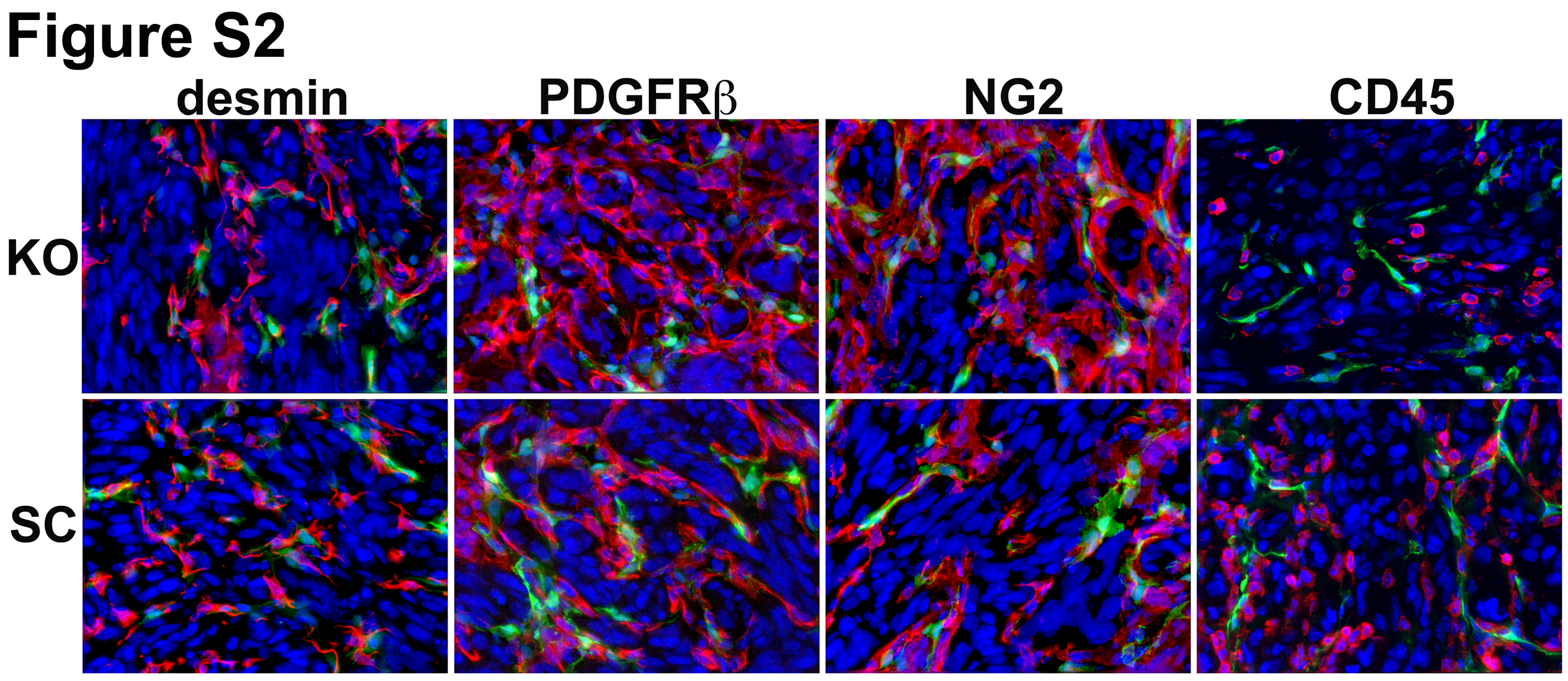

Supplement: Figure S2 — Tumor Ad5ROBO4-EGFP expression is endothelial cell restricted. 786-O kidney orthotopic (KO) and subcutaneous (SC) xenograft expression of the pericyte and stromal fibroblast markers desmin, PDGFRβ, and neural glial antigen-2 (NG2) is contained within a tissue compartment distinct from EGFP expressing cells. Similarly CD45 cells lack EGFP expression. Magnification: 200X. Red: each stromal protein; Green: EGFP immunofluorescence; Blue: DAPI. (TIF) [file pone.0083933.s002.tif]

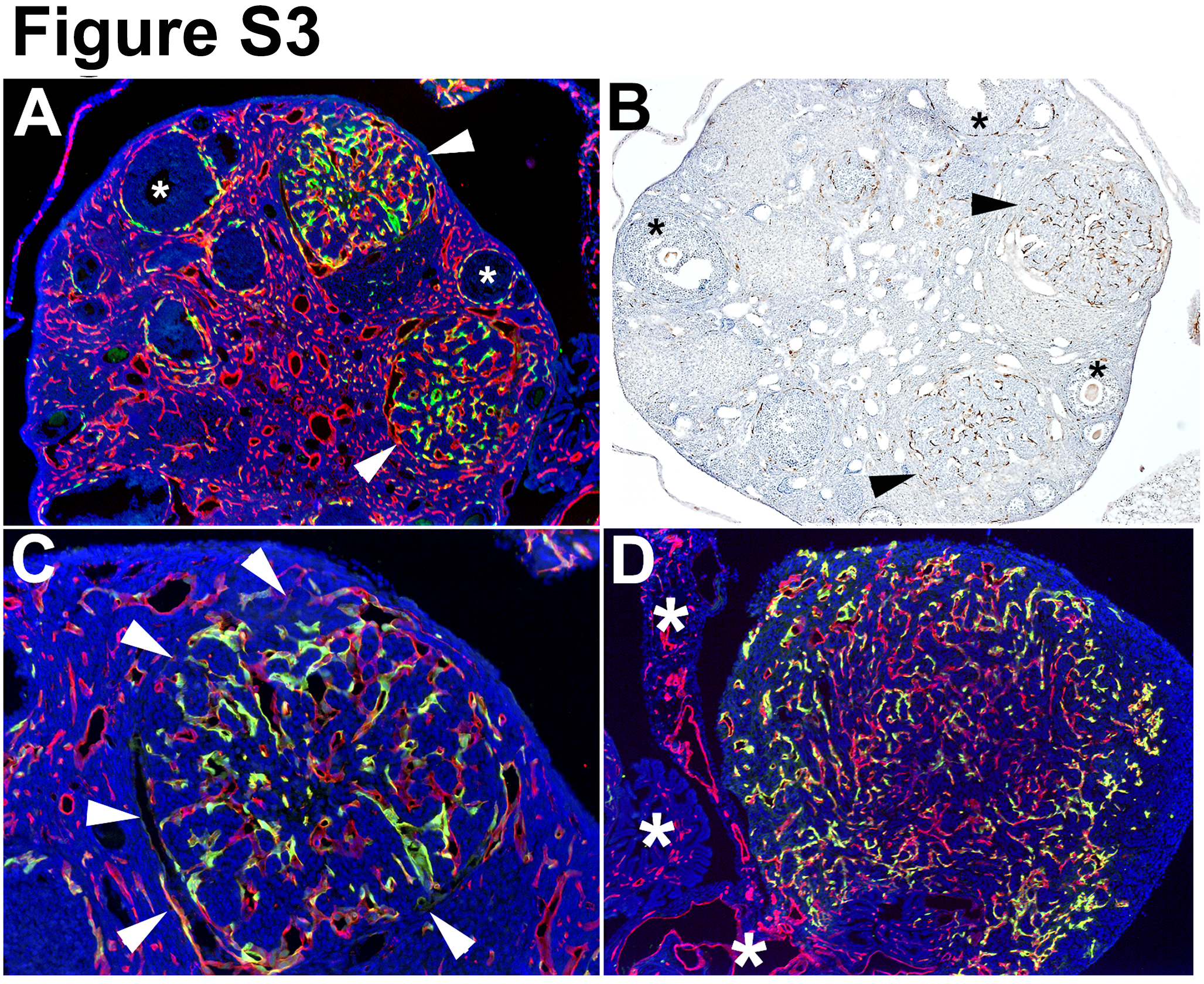

Supplement: Figure S3 — Ad5ROBO4 transcriptionally targets metastatic endothelium. A–D. Intra-, and peri-ovarian “Krukenberg” renal carcinoma metastases from subcapsular 786-O orthografts in hCAR:Rag2−/− mice injected with 1.5×10^11^ vp display extensive and intense microvessel EGFP immunofluorescence. A. and B. Nearly all vessels within the two ovarian micrometastases (arrowheads) express the Ad5ROBO4 vector, whereas EGFP immunofluorescence is only detected in circumferential microvessels immediately adjacent to host ovarian follicles (asterisks), but not in stromal microvessels. C. Higher magnification view of one of the metastases revealing near ubiquitous intratumoral Ad5ROBO4 vector vascular expression. D. Near ubiquitous Ad5ROBO4 vascular expression is also evident in microvessels within a peritoneal metastasis adherent to the adjacent host fallopian tube (asterisks) whose vessels are negative for vector expression. Magnification: A and B 40X, C 200X, D 100X. A, C and D: Red: endomucin/CD31 cocktail, Green: EGFP immunofluorescence, Blue: DAPI. B: EGFP immunohistochemistry (brown) and hematoxylin counterstain. (TIF) [file pone.0083933.s003.tif]

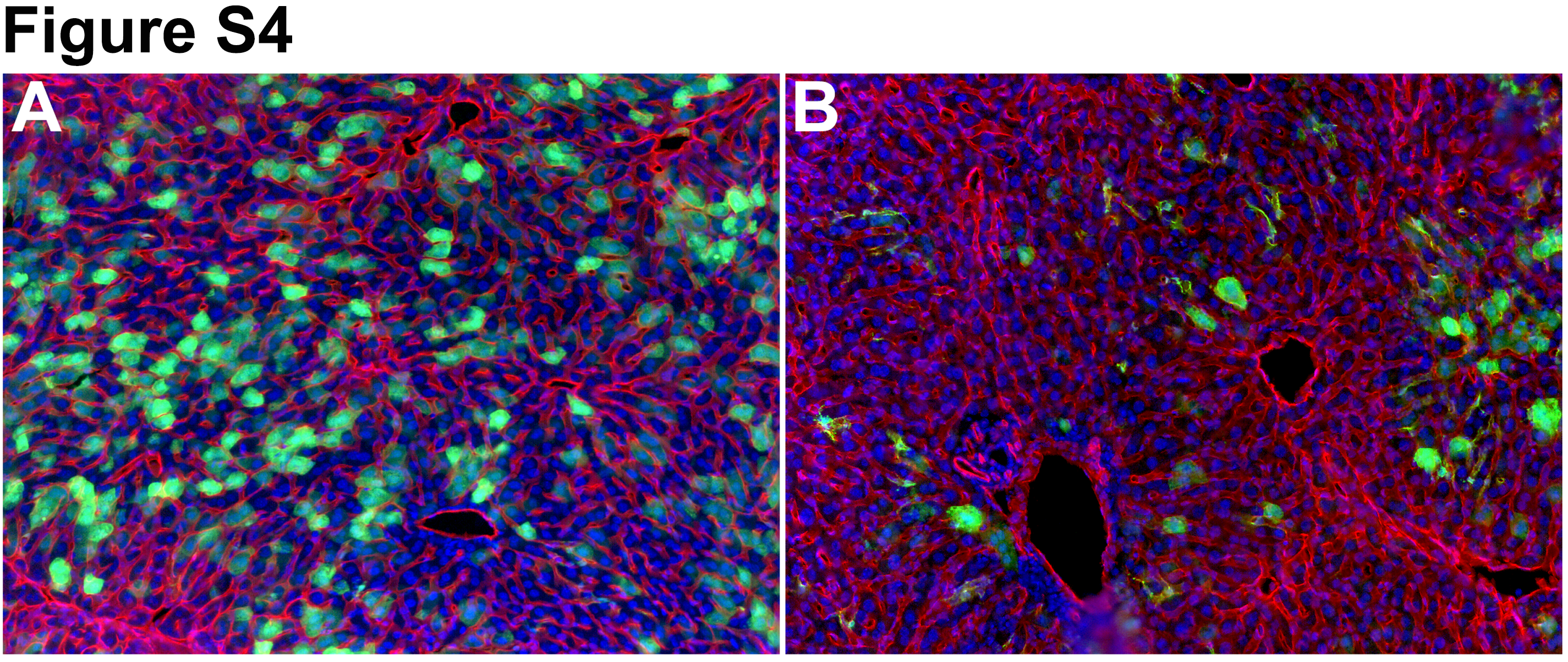

Supplement: Figure S4 — Large format view of warfarin mediated liver detargeting. A. Vehicle (peanut oil) pretreated Rag2−/− mice injected with 1.0×10^11^ vp of Ad5CMV evidence EGFP expression localized to liver hepatocytes. B. Warfarin pretreatment markedly decreased the frequency of positive hepatocyte expression of the Ad5CMV vector, while producing sporadic expression in reticuloendothelial system and rare endothelial cells. Magnification: 100X. Red: endomucin/CD31 cocktail, Green: EGFP immunofluorescence, Blue: DAPI. (TIF) [file pone.0083933.s004.tif]

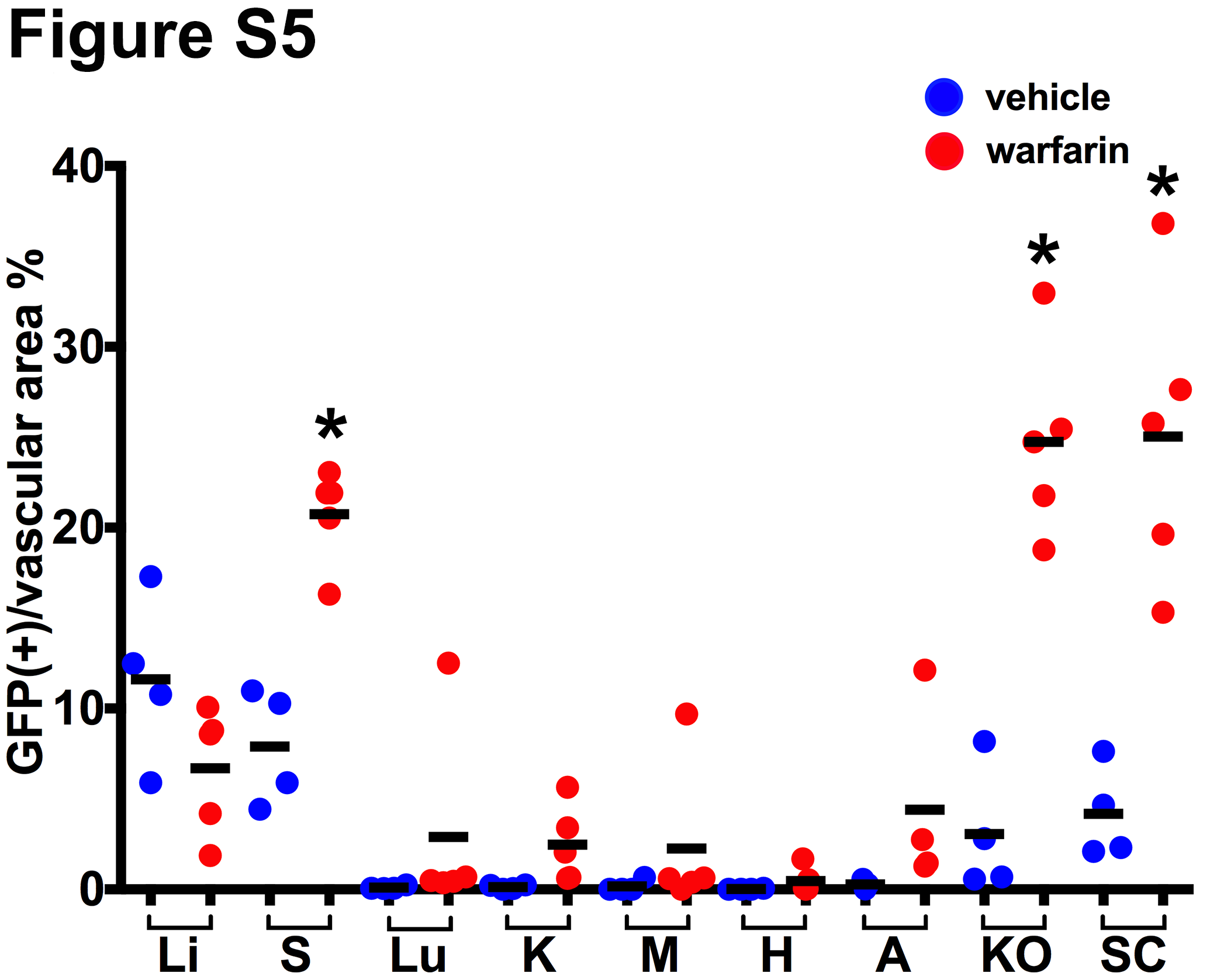

Supplement: Figure S5 — Warfarin liver detargeting markedly increases intratumoral and splenic EGFP positive vascular areas without significant host organ expression. Ratios of tissue section areas positive for EGFP colocalized with CD31/endomucin immunofluorescence over total CD31/endomucin immunofluorescence determined using image analysis software. In vehicle treated mice (n = 4), liver, spleen, kidney orthotopic and subcutaneous tumors are the only vascular beds with an appreciable extent of Ad5ROBO4 endothelial cell expression. Warfarin (n = 5 mice), mediated a marked enhancement of the extent of vector expressing tumoral vascular areas with a decrease in liver vascular area vector expression. Splenic vector positive area also appreciably increased with barely detectable induction in all other host organs except for a single outlier mouse. Blue dots: mean of four 100X fields for each vehicle-treated mouse, Red dots: mean of four 100X fields for each warfarin-treated mouse. *p<0.05 one-way ANOVA with Tukey's multigroup correction comparing vehicle and warfarin. (TIF) [file pone.0083933.s005.tif]
